# An fMRI investigation of delay discounting in patients with schizophrenia

**DOI:** 10.1002/brb3.135

**Published:** 2013-04-24

**Authors:** Kathy Burton Avsar, Rosalyn Eve Weller, James Edward Cox, Meredith Amanda Reid, David Matthew White, Adrienne Carol Lahti

**Affiliations:** 1 Department of Psychiatry and Behavioral Neurobiology, The University of Alabama at BirminghamBirmingham, Alabama, 35294-0017; 2 Department of Psychology, The University of Alabama at BirminghamBirmingham, Alabama, 35294-1170; 3 Department of Biomedical Engineering, The University of Alabama at BirminghamBirmingham, Alabama, 35294-2182

**Keywords:** Delay discounting, executive function, intertemporal, reward, schizophrenia, subjective value

## Abstract

Schizophrenia (SZ) is associated with a reduced ability to set meaningful goals to reach desired outcomes. The delay-discounting (DD) task, in which one chooses between sooner smaller and later larger rewards, has proven useful in revealing executive function and reward deficits in various clinical groups. We used fMRI in patients with SZ and healthy controls (HC) to compare brain activation during performance of a DD task. Prior to the neuroimaging session, we obtained each participant's rate of DD, *k*, on a DD task and used it to select a version of the DD task for each participant's fMRI session. Because of the importance of comparing fMRI results from groups matched on performance, we used a criterion value of *R*^2^ > 0.60 for response consistency on the DD task to analyze fMRI activation to DD task versus control trials from consistent SZ (*n* = 14) and consistent HC (*n* = 14). We also compared activation between the groups on contrasts related to trial difficulty. Finally, we contrasted the inconsistent SZ (*n* = 9) with the consistent HC and consistent SZ; these results should be interpreted with caution because of inconsistent SZ's aberrant performance on the task. Compared with consistent HC, consistent SZ showed reduced activation to DD task versus control trials in executive function and reward areas. In contrast, consistent SZ showed more activation in the precuneus and posterior cingulate, regions of the default mode network (DMN) that are typically deactivated during tasks, and in the insula, a region linked to emotional processing. Furthermore, consistent SZ had abnormal activation of lateral and medial frontal regions in relation to trial difficulty. These results point to disruption of several neural networks during decision making, including the executive, reward, default mode, and emotional networks, and suggest processes that are impaired during decision making in schizophrenia.

## Introduction

Schizophrenia (SZ) is a heterogeneous disorder, with patients exhibiting a wide range of symptoms and functional outcomes. The positive symptoms of delusions and hallucinations are typically most prominent and used in the diagnosis. However, it is cognitive and motivational deficits that contribute most to poor functional outcomes (Niendam et al. 2006). In contrast to positive symptoms, these deficits are not improved by treatment (Green 1996). Deficit in motivation and drive leading to impaired decision making is a core feature of SZ. Recent studies have shown that while patients with SZ experience pleasure in response to positive stimuli (“liking”) to the same extent as healthy volunteers (HC), their ability to experience anticipatory pleasure (“wanting”), and thus to initiate goal-seeking behaviors is impaired (Barch and Dowd 2010). Drawing on the field of affective neuroscience, Barch and Dowd (2010) recently proposed a brain network–based model that integrates the processes encompassing decision making. These processes, which include attribution of hedonic value (liking), reward prediction (wanting), cost–benefit analysis, and action plan toward valued outcome, are subserved by distinct but overlapping brain networks. The integration of these neural networks, including the executive and reward networks, is necessary to achieve optimal decision making.

Widespread neural abnormalities, both morphological (Harrison 1999) and functional (Minzenberg et al. 2009), have been reported in SZ in regions associated with executive function, such as prefrontal and parietal cortex (Perlstein et al. 2001; Callicott et al. 2003; Manoach 2003; Tan et al. 2007), and with reward processing, such as ventral striatum and midbrain (Juckel et al. 2006a,b; Jensen et al. 2008; Murray et al. 2008; Schlagenhauf et al. 2008; Waltz et al. 2009; Koch et al. 2010; Romaniuk et al. 2010). Understanding how neural abnormalities may disrupt the integration of information between executive function and the reward system offers a window into better understanding of the functional deficits in SZ.

Decision making requires choosing between alternative behaviors that may require short-term sacrifice for long-term gain, similar to choices in the laboratory delay-discounting (DD) task. The DD task requires a series of choices between receiving a small sooner (usually immediate) reward or a larger delayed reward (DR) (Rachlin et al. 1991; Green et al. 1996). Greater willingness to wait for larger but later rewards, or smaller DD, has been associated with less impulsivity (Ainslie 1975) and better cognition and executive function (Shamosh et al. 2008). Individuals with various addictions (Vuchinich and Simpson 1998; Bickel et al. 1999; Kirby et al. 1999; Mitchell 1999; Petry and Casarella 1999; Baker et al. 2003; Robles et al. 2011) and some psychiatric conditions (Crean et al. 2000; Petry 2001; Takahashi et al. 2008) show greater DD than controls do.

Most studies using the DD paradigm characterize an individual's choices by generating a discount function for him or her that models the effect of delay on subjective value of later rewards (e.g., Bickel et al. 1999; Heerey et al. 2007; Kirby et al. 1999). The parameter *k* is the rate at which an individual discounts future rewards, with larger *k*'s indicating greater DD (Mazur and Coe 1987; Rachlin et al. 1991). However, few studies have systematically investigated choice or response consistency in DD. Consistency is highly relevant to SZ, as many studies have noted that inconsistency of behavior and performance is one of the notable features of SZ (Cohen et al. 1999; Schooler et al. 2008). Often *R*^2^ has been used to index degree of consistency, that is, the correspondence between data points and a mathematical discounting model (for review and other methods of defining consistency, see Johnson and Bickel 2008).

Previous behavioral studies of DD in SZ have yielded mixed results (Heerey et al. 2007, 2008, 2011; MacKillop and Tidey 2011; Wing et al. 2012; but see Ahn et al. 2011). Heerey and Gold (2007) and Heerey et al. (2011) reported greater DD in SZ and showed that greater DD was correlated with worse working memory, suggesting that, in SZ, greater DD was related to an inability to represent future outcomes rather than an inability to delay gratification. Because the incidence of smoking is very high in SZ (Hughes et al. 1986; Kalman et al. 2005; de Leon and Diaz 2005) and smokers show greater DD than nonsmokers (Bickel et al. 1999; Baker et al. 2003), two recent studies evaluated the effect of smoking on DD in SZ; they found *no* group differences in DD between SZ and healthy controls (HC) (MacKillop and Tidey 2011; Wing et al. 2012; but see Ahn et al. 2011).

A number of studies have investigated DD using functional magnetic resonance imaging (fMRI; e.g., McClure et al. 2004; Kable and Glimcher 2007; Weber and Huettel 2008; Marco-Pallares et al. 2010). Although the neural substrates of DD are debated, DD trials in general activate a broad putative decision making network (McClure et al. 2004; Hoffman et al. 2006; Monterosso et al. 2007; Bickel et al. 2009; Pine et al. 2009). McClure et al. (2004) suggested that all DD trials and, in particular, more difficult decisions, are subserved by the frontoparietal system, whereas immediate choices are mediated by the limbic system. There has been no prior fMRI study of DD in SZ.

The main goal of this study was to determine whether the neural correlates of DD were abnormal in SZ compared with HC. A key feature of our design was to match groups as closely as possible on task performance. We have found this consideration to be important in studying individuals with SZ (Avsar et al. 2011). In this and a previous study (R. E. Weller, K. B. Avsar, J. E. Cox, M. A. Reid, D. M. White, A. C. Lahti, unpubl. ms.), a substantial percentage of the SZ group exhibited aberrant performance on DD, suggesting inability to perform the task or lack of engagement on the task. Including such participants in an fMRI analysis would potentially make group differences in brain activation impossible to interpret. Data from such participants were therefore excluded from the main group comparisons. The resulting HC and SZ groups (*n* = 14 in each) were well matched on both DD response consistency and rate of DD. We believe that the benefits of our matching strategy in terms of interpretability of the fMRI results outweigh the possible loss of generality from excluding so many SZ. However, for the sake of completeness, we also provide the imaging results for the inconsistent SZ.

We first investigated activation to all DD task trials compared with sensorimotor control (SMC) trials, a contrast tapping into the broad decision making process. We hypothesized that SZ compared with HC would show less activation in regions of the executive and reward networks. In addition, we investigated activation on difficult trials and easy trials; contrasts thought to invoke the executive function network during the more difficult trials and limbic regions during the easy trials (McClure et al. 2004; Monterosso et al. 2007; Marco-Pallares et al. 2010). On the basis of known literature (Perlstein et al. 2001; Callicott et al. 2003; Manoach 2003; Tan et al. 2007), we hypothesized SZ would demonstrate less activation in prefrontal regions during the difficult trials.

## Material and Methods

### Participants and assessments

Participants were 56 individuals recruited from the University of Birmingham (UAB) area. Thirty-five of these participants were patients with DSM-IV (American Psychiatric Association. American Psychiatric Association. Task Force on DSM-IV 2000) schizophrenia or schizoaffective disorder (SZ), diagnoses established using patients’ medical records and the Diagnostic Interview for Genetic Studies (Nurnberger et al. 1994), and recruited from UAB outpatient psychiatric clinics. Twenty-one HC were recruited from the community using flyers and advertisements in the University newspaper. Common exclusion criteria were major medical conditions, substance abuse within the past 6 months, previous serious head injury, a neurological disorder, previous loss of consciousness, pregnancy, or ferromagnetic material in the body. HC were also excluded for any current or lifetime significant (e.g., depression, anxiety) Axis I diagnosis. The study was approved by the Institutional Review Board of the University of Alabama at Birmingham, and all participants gave written informed consent. The study was conducted in compliance with the standards established by UAB's Institutional Review Board and with the Code of Ethics of the World Medical Association. Participants received compensation between $92 and $99, depending on performance on an unrelated task in the magnet.

We used the Repeatable Battery of Neuropsychological Status (RBANS) (Randolph et al. 1998) to measure general cognitive function in all participants and the Brief Psychological Rating Scale (BPRS) (Overall and Gorham 1962) in patients to measure positive (conceptual disorganization, hallucinatory behavior, and unusual thought content) and negative (emotional withdrawal, motor retardation, and blunted affect) mental status and symptoms (See Table 1 and Table S4 for demographic characteristics and cognitive and behavioral assessments for patients and controls).

**Table 1:** Demographic data and clinical and behavioral measures for participants used in fMRI analyses

Variable	Healthy controls (*n* = 14)	Patients (*n* = 14)	*P*
Age	34.07 ± 2.89	36.50 ± 3.52	0.60
Gender	8 men; 6 women	10 men; 4 women	0.43
Parental socioe-conomic status^1^	6.10 ± 4.14	5.92 ± 1.30	0.93
Smoking^2^/Number of Smokers	0.27 ± 0.11/5	0.59 ± 0.15/10	0.06^3^/0.06
Age of onset		19.14 ± 2.47	
Duration^4^ (years)		17.36 ± 3.33	
Antipsychotic medication^5^		1st-1, 2nd-12, C-1	
RBANS^6^			
Total index	95.69 ± 3.01	77.93 ± 2.81	<0.001
Immediate memory	97.15 ± 3.10	82.64 ± 3.58	0.005
Visuospatial	96.62 ± 4.35	80.50 ± 4.96	0.023
Language	96.46 ± 4.06	91.36 ± 1.90	0.25
Attention	98.46 ± 4.58	83.43 ± 4.35	0.025
Delayed memory	98.00 ± 1.73	77.79 ± 5.13	0.002
BPRS			
Total		32.57 ± 2.61	
Positive		6.64 ± 1.06	
Negative		4.93 ± 0.61	
Delay discounting			
Log of Imaging *k*	−1.91 ± 0.18	−1.70 ± 0.18	0.40
Imaging *R*^2^	0.92 ± 0.01	0.91 ± 0.02	0.95

Values are means ± standard error except where noted. RBANS, Repeatable Battery of Neuropsychological Status; BPRS, Brief Psychological Rating Scale.

1 Data not available for four healthy controls and one patient.

2 Packs per day (20 cigarettes = one pack).

3 Result of Mann–Whitney test.

4 Data not available for one SZ.

5 1st, first generation; 2nd, second generation; C, combination.

6 RBANS data not available for one healthy control.

### Delay-discounting tasks

**Figure 1:**
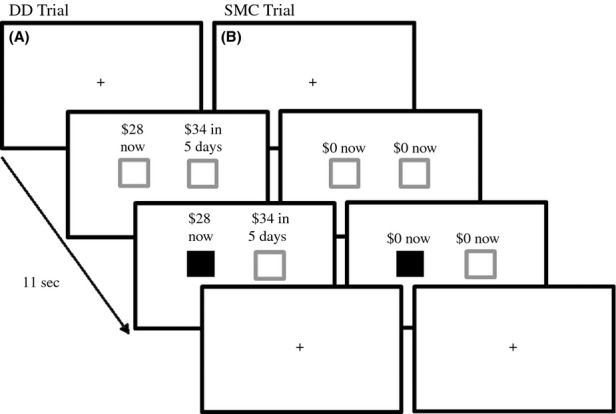
Delay-discounting (DD) task. (A) DD task trial; (B) sensorimotor control (SMC) trial. All trials were 11 sec in duration, with the initial fixation cross presented for 2, 4, or 6 sec, followed by two gray boxes paired with (A) the choice of an immediate or a delayed hypothetical monetary reward ($28 now or $34 in 5 days; a trial *k* of 0.041) or (B) the no-choice option. Participants had the remainder of the 11 sec trial (9, 7, or 5 sec) to indicate their preference by pressing a button on the side corresponding to their choice. The box under the choice turned green, indicating the response selection. A return of the fixation cross indicated the start of the next trial.

We first tested participants in the laboratory on a DD task, modified from Kirby and colleagues (Kirby et al. 1999; Kishinevsky et al. 2012). Participants viewed the 108 trials of the laboratory DD task on a computer monitor; 96 trials were divided equally between eight categories with differing trial *k* values, interspersed with 12 SMC trials, for which participants arbitrarily made a right or left button response (Fig. 1). Each trial consisted of a choice between a unique combination of an immediate reward (IR), ranging from $1 to $73, and a DR, ranging from $28 to $86, with delays (D) ranging from 1 to 116 days. All rewards were hypothetical. Choices were generated for the eight trial *k*'s by adjusting reward values and D using the hyperbolic function, IR = DR/(1 + *kD*) (Mazur and Coe 1987). From the results, each participant's rate of discounting (*k*) was estimated (see below), allowing us to choose an appropriate DD task to use during imaging such that the participant would be expected to select about half immediate and half delayed choices (see Data S1, Methods).

The scanning session took place immediately after the laboratory session. The magnet DD task was identical to the laboratory DD task except for the number of trials and distribution of trial *k*'s. Each of 10 possible magnet tasks included five trial categories based on trial *k*'s (*k*_1_–*k*_5_; see Data S1, Table S1). Based on the laboratory results, a magnet task was chosen with a *k*_3_ (middle trial *k* value) nearest to the participant's *k*. The three trial categories with trial *k* nearest to the participant's *k* (*k*_2_–*k*_4_) are referred to as difficult trials because the subjective values of the immediate and DRs would be similar. Overall for difficult trials, percentages of immediate and delayed choices were approximately equal (Marco-Pallares et al. 2010). The *k*_1_ and *k*_5_ trial categories are referred to as easy trials because the subjective values of the immediate and DRs were assumed to be dramatically different; greater for IRs on *k*_1_ trials, with immediate choices predominating, and greater for DRs on *k*_5_ trials, with delayed choices predominating. The magnet task consisted of four 7:24 min runs, each with 30 task trials divided equally between the five trial *k* values and 10 SMC trials. Thus, the magnet DD task was more difficult than the laboratory DD task because 3/5 (*k*_2_–*k*_4_) of the trials were difficult or relatively difficult, and because the magnet DD task consisted of more trials. Stimuli were projected onto a mirror mounted on the head coil, using IFIS-SA (MRI Devices, Waukesha, WI).

### Imaging data acquisition

Scans were acquired using a Siemens Allegra head-only 3T magnet (Erlagen, Germany) with a single-channel circularly polarized no-tune transmit/receive head coil. For BOLD (blood oxygen level–dependent) fMRI scans, an echo planar imaging sequence with a 2.2 sec repitition time (TR), 30 msec echo time (TE), and 70° flip angle was used to acquire 30 interleaved 4.0 mm axial slices (1 mm gap). The field of view was 24 × 24 cm^2^. These acquisition parameters resulted in 3.8 × 3.8 × 4.0 mm voxels. We also acquired a high-resolution anatomical T1-weighted image using a MPRAGE sequence. E-Prime software (version 1.2; Psychology Software Tools, Pittsburgh, PA) running on an IFIS-SA system was used to control stimulus delivery and record responses and reaction times.

### Data analysis

#### Calculation of *k* and *R*^2^

We entered percentages of immediate choices (%Now) and corresponding trial *k* values into nonlinear (exponential) regression analysis to determine each participant-specific *k*, defined as a trial *k* value corresponding to predicted %Now or %Immediate choices = 50%. The analysis also yielded a model fit statistic, *R*^2^, used as an index of consistency that provided a measurement of how well the participant's responses fit the expected pattern of decreasing preferences for the IR as trial *k* increased.

#### Delay-discounting behavioral measures

Independent samples *t* tests were used to compare the groups of HC and SZ (see Results) on rate of discounting and response consistency from the imaging session. For the former, we compared log_10_(*k*) because distributions of *k* are severely skewed (Johnson and Bickel 2002; Heyman and Gibb 2006). For the latter, *R*^2^ values were transformed to Fisher's *R*’ values (Howell 2007). Similar analyses were used to compare groups on age, parental socioeconomic status (SES), RBANS scales, and BPRS scales. For comparison of packs of cigarettes smoked per day, the nonparametric Mann–Whitney test was used because of the high frequency of 0 values, resulting in positively skewed distributions. Gender composition of groups was compared using the *χ*^2^ test of independence. Finally, mixed between- and within-group ANOVA was performed to compare groups on %Now and response time (RT) across trial *k* values. Holm's procedure (Howell 2007) was used to correct for multiple comparisons in follow-up analyses. For all analyses, *α* = 0.05.

#### Imaging data analysis

Image preprocessing was carried out using SPM8 (http://www.fil.ion.ucl.ac.uk/spm/software/spm8/) in MATLAB. Slices were corrected for differences in acquisition times using slice time correction. Participant movement was corrected using a least squares method of realignment, and ArtRepair (Mazaika et al. 2007) was used to correct movement artifacts by interpolating between slices when movement exceeded 0.5 mm per TR. Data greater than 2-mm movement per 40 trial runs were not used in analyses. Each participant's anatomical scan was coregistered to the SPM canonical MNI template (Montreal Neurological Institute, Montreal, Canada) initially using linear body registration, then normalized using the diffeomorphic image registration algorithm model (Ashburner 2007) to produce a flow field. The flow field was then used to normalize functional images that were registered to each participant's anatomical image in MNI space.

Statistical analysis of the preprocessed functional images was conducted for each participant using the General Linear Model (Dickey et al. 2010) to detect areas where changes in BOLD response were correlated with stimulus presentation and response. Individual responses were modeled using a variable epoch approach (Grinband et al. 2008); RT for trials was modeled using a boxcar epoch, with onset vectors corresponding to stimulus presentation and duration equal to latency to button push. Trials were divided into two conditions based on trial difficulty (hard, easy). In addition, vectors that corresponded to the onset of SMC trials were included in the design matrix. Each regressor of interest was convolved with the canonical hemodynamic response function followed by a time derivative. Cognitive subtraction (Price and Friston 1997; Nichols et al. 2005) was used to contrast brain activation to all DD task trials>SMC trials, hard>easy trials, and easy>hard trials to produce statistical parametric contrast images to be carried into second-level analyses. We also included analyses of hard trials>baseline and easy trials>baseline (baseline being the period a fixation cross appeared between each pair of choices) for between-group comparisons. Individual-participant general linear models were created to estimate parameters for the contrasts of task trials versus SMC trials and contrasts related to difficulty. One-way repeated-measures ANOVAs were used for within-group analyses (consistent SZ and consistent HC). Subsequently, groups were compared using a two-way (group *x* trial category) ANOVA. Cluster size was defined as the number of contiguous voxels for which *P* < 0.05, uncorrected, except for within-group contrasts of task>SMC for which *P* < 0.001, uncorrected, for all voxels in a cluster. Cluster-size threshold was defined within SPM8 on the basis of Gaussian random-field theory to maintain the false discovery rate (FDR) = 0.05 (Genovese et al. 2002; Chumbley and Friston 2009).

## Results

### Behavioral and clinical assessments

**Figure 2:**
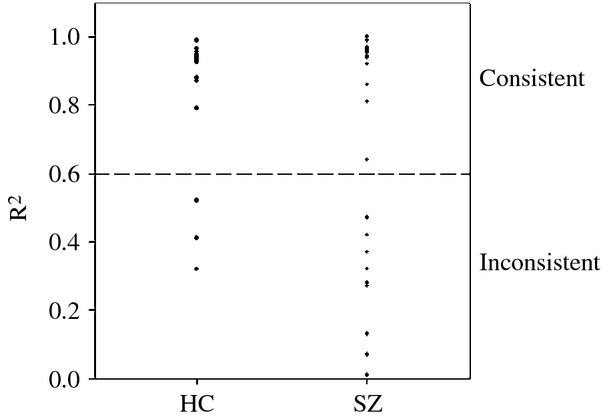
Individual model fit (*R*^2^) values during estimation of *k* values for healthy controls (HC) and patients with schizophrenia (SZ). The line at 0.60 indicates the minimum *R*^2^ value that was used to define consistent performance.

When data from the magnet DD task were analyzed, the resulting distribution of *R*^2^ values from SZ participants was approximately bimodal (Fig. 2), with a majority showing high values and the remainder showing very low values, suggesting an inability to make consistent choices. Three HC also had low *R*^2^ (Fig. 2). As we did in a previous study (R. E. Weller, K. B. Avsar, J. E. Cox, M. A. Reid, D. M. White, A. C. Lahti, unpubl. ms.), we set a criterion of *R*^2^ > 0.60 to define consistent performance in order to analyze fMRI data from HC and SZ matched on consistent performance on the DD magnet task. As described below, this also resulted in the groups being very similar on rate of discounting.

Overall, data from seven of 21 controls were not included in the imaging analyses: in addition to the three who were inconsistent on the DD task, two exceeded movement criteria, one did not respond to the SMC trials, and one was unable to tolerate the scanner (i.e., was claustrophobic). Data from 16 of 35 SZ were excluded based on performance on the magnet DD task: nine on the basis of *R*^2^ < 0.60 and seven because their responses were nearly all (>85%) choices of either immediate or DRs, suggesting lack of engagement in the decision making task. In addition, three patients exceeded movement criteria and two were unable to tolerate the scanner, leaving a group of 14 consistent SZ for analyses. For completeness, we include the behavioral results and results of fMRI activation to task versus SMC trials for the inconsistent SZ compared with the other two groups, although we recognize that the performance confounds in such fMRI data make their interpretation ambiguous.

**Figure 3:**
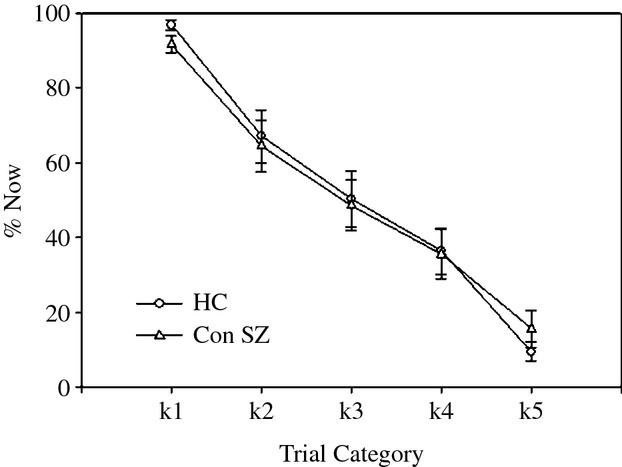
Mean (± standard error) for percentage of Now (%Now) choices as a function of the five trial *k*'s for the consistent HC and consistent patients with schizophrenia (Con SZ).

Demographic characteristics and cognitive and behavioral assessments (Nurnberger et al. 1994; Randolph et al. 1998) for the consistent SZ and HC are shown in Table 1. Of the 14 consistent controls and 14 consistent patients used in the imaging analyses, the groups were well matched with regard to consistency and rate of discounting; differences in *R*’ and log_10_(*k*) did not approach significance. Figure 3 shows that both groups reduced the percentage of IR choices to a similar degree as trial *k* values increased. Neither the main effect of Group (*F*[1,26] = 0.018, *P* = 0.89) nor the Group *x* Trial *k* interaction (*F*[4104] = 0.54, *P* = 0.71) was significant; nor were there significant group differences at individual trial *k*'s.

**Figure 4:**
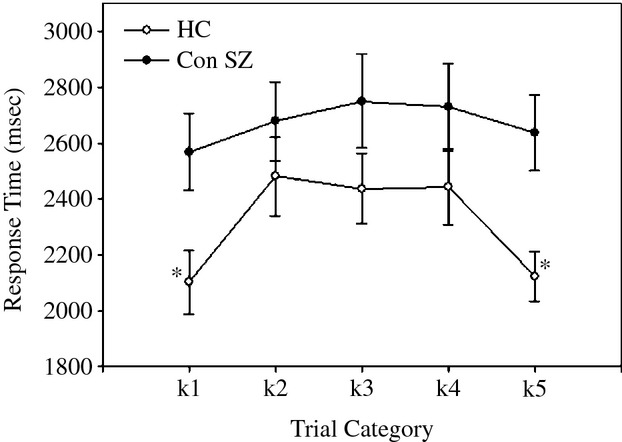
Mean (± standard error) of response times across the five trial categories during the scanning session for the consistent healthy controls (HC) and consistent patients (Con SZ). *P < 0.05 between groups.

The graph of mean RT across trial *k*'s for HC showed a distinct inverted-U shape (Fig. 4). ANOVA revealed a significant effect of Trial Category (*F*[4,52] = 7.65, *P* < 0.001), as well as a significant quadratic trend (*F*[1,13] = 13.85, *P* = 0.003). In subsequent contrasts of easy versus difficult trials (*k*_1_ vs. *k*_2_–*k*_4_ and *k*_5_ vs. *k*_2_–*k*_4_), RT for easy trials was significantly shorter than for difficult trials (*P* values <0.025). By contrast, consistent SZ did not significantly modulate RT among trials (*F*[4,52] = 1.07, *P* = 0.38). ANOVA comparing groups revealed a significant effect of Group (*F*[1,26] = 4.32, *P* = 0.048) but no significant Group *x* Trial Category interaction (*F*[4104] = 1.81, *P* = 0.13). RT was generally longer in SZ compared with HC. Figure 4 suggests that this effect across trial *k*'s tended to be most pronounced for easy trials (*k*_1_ and *k*_5_). SZ also responded more slowly on SMC trials than HC did (means = 1226 vs. 863 msec, respectively, *t*[26] = 5.39, *P* < 0.001).

As seen in Table 1, the consistent SZ and HC groups did not differ significantly on age, gender, or parental SES, which is important because some demographic characteristics such as age and income have been found to be related to greater DD (Green et al. 1996; Samanez-Larkin et al. 2011). The group difference in smoking was marginally significant (*P* = 0.06), with patients smoking more than controls. Smoking is related to a higher rate of DD (Bickel et al. 1999; Baker et al. 2003), but this possible confound was not an issue as we did not find a difference in rate of discounting between consistent SZ and consistent HC. Consistent SZ scored significantly worse than HC on all RBANS scales except for language. It should be noted that laboratory DD results, demographic characteristics, and cognitive assessments for all imaging participants of this study (except one inconsistent SZ) were included in the larger samples of HC and SZ in R. E. Weller, K. B. Avsar, J. E. Cox, M. A. Reid, D. M. White, A. C. Lahti (unpubl. ms).

**Figure 5:**
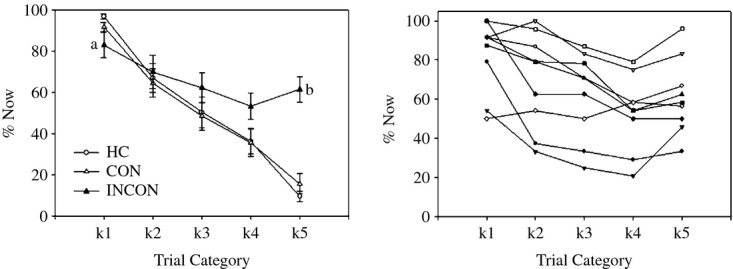
Mean (± standard error) percentage of Now (%Now) choices as a function of the five trial *k*'s for the consistent healthy controls (HC), consistent SZ, and inconsistent SZ (INCON) (left) and for individual inconsistent SZ (*n* = 9; right). a, *P* = 0.005 for inconsistent SZ versus HC and 0.07 versus consistent SZ; b, *P* < 0.001 for inconsistent SZ versus HC and versus consistent SZ.

The inconsistent SZs for whom quality scans were available (*n* = 9) did not differ from the consistent SZ group on any of the demographic variables, BPRS or RBANS scores, with the exception of the Delayed Memory score of the RBANS where inconsistent SZ scored worse than Consistent SZ (Table S4). By definition, inconsistent SZ had lower *R*^2^ (mean = 0.26) than consistent SZ and HC but also significantly higher log(*k*) (mean = −0.019). However, the validity of the computed *k* values for this group is suspect. Inspection of Figure 5, left, reveals that percentage of Now responses (%Now) by inconsistent SZ was significantly higher for *k*_5_ trials than for the other groups, consistent with greater DD, but %Now was also significantly lower versus HC for *k*_1_ trials, contrary to what would be expected for more impulsive individuals. Furthermore, %Now values from individual inconsistent SZ (Fig. 5, right) reveal that a large percentage failed to show the expected pattern of decreasing %Now as trial *k*'s increased, suggesting that the task was too difficult and/or that the participants were not meaningfully engaged in the task.

It should be noted that in our behavioral study of performance on the laboratory version of the DD task (R. E. Weller, K. B. Avsar, J. E. Cox, M. A. Reid, D. M. White, A. C. Lahti, unpubl. ms.), almost all (21/23) the SZ participants in this study had met the same criterion level of consistency used in this study, *R*^2^ > 0.60. However, the laboratory task is undoubtedly easier than the magnet task, consisting of fewer trials and with a higher percentage of easy trials, on which the subjective values of the two choices are markedly different. In addition, participants often find the magnet environment to be stressful. The inability of approximately 40% of previously consistent SZ to appropriately perform the task in the magnet may be attributable to this combination of greater difficulty and stress (Mazure 1995). Those SZ who switched from consistent in the laboratory to inconsistent in the magnet had significantly lower *R*^2^, as well as higher log(*k*) in the laboratory session than those who remained consistent. Thus, it was the originally marginal performers who were subsequently unable to perform the task under more challenging conditions.

### Imaging results

#### Consistent groups

**Figure 6:**
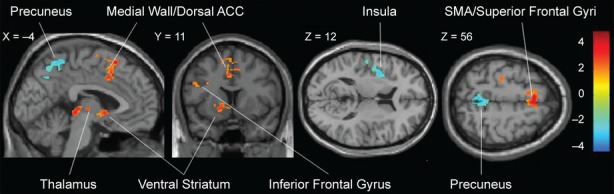
Between-group results for activation to task>SMC trials revealed more activation in controls (red) in frontoparietal areas, including inferior frontal gyrus and medial areas of the prefrontal cortex, and subcortically in the striatum and thalamus; *x*, *y*, and *z* are MNI coordinates. Patients (blue) had more activation than controls in the precuneus and insula. *P* < 0.05, false discovery rate (FDR) corrected for cluster extent. Bar at right represents *t* values.

The first comparison of interest was activation to all DD task trials versus SMC trials. In the within-group results, consistent HC qualitatively showed more widespread activation, such as in putative executive function areas (the inferior and middle frontal gyri, dorsal anterior cingulate cortex or dACC, and inferior parietal lobule), attention-related areas (precuneus), and midbrain, to the task than did consistent SZ (Table S2, Fig. S1). In the consistent between-group analysis (Table 2, Fig. 6), significantly enhanced activation in DD over SMC trials in the HC (Fig. 6, red) occurred in regions including the inferior frontal gyrus; medial wall locations such as dACC extending into supplementary motor area (SMA) and pre-SMA motor areas; posterior parietal cortex extending into occipital cortex; and subcortically, in the ventral striatum, thalamus, and midbrain. By contrast, greater activation in the SZ group (Fig. 6, blue) was found in the insula, with the cluster extending into the frontal operculum and superior temporal gyrus, and in a more posterior medial wall cluster that included the precuneus and posterior and middle cingulate gyrus.

**Table 2:** Consistent patients and consistent controls: between-group fMRI results for DD task>SMC trials^1^

Brain regions^2^	Cluster^3^	Voxels^4^	*x*^5^	*y*^5^	*z*^5^	*t*	*P*^6^
Controls(task>SMC)>patients(task>SMC)							
Frontal cortex – L	1366		−48	16	29	3.98	0.015
Inferior frontal gyrus		289					
Parietal/Occipital cortex – R	1161		30	−50	47	4.98	0.022
Inferior parietal gyrus		428					
Angular gyrus		132					
Supramarginal gyrus		52					
Precuneus		72					
Superior occipital gyrus		133					
Parietal/Occipital cortex – L	1651		−42	−41	48	3.70	0.007
Superior parietal gyrus		234					
Inferior parietal gyrus		821					
Angular gyrus		140					
Middle occipital gyrus		172					
Medial Wall/Dorsal ACC	1298		2	18	59	3.66	0.015
Supplementary motor area – R^7^		538					
Supplementary motor area – L^7^		375					
Middle cingulate gyrus – R		56					
Middle cingulate gyrus – L		111					
Thalamus/Basal ganglia	1627		4	3	0	3.60	0.007
Thalamus – L		255					
Thalamus – R		247					
Ventral striatum – L		249					
Pallidum – L		69					
Midbrain		360					
Patients(task>SMC)>controls(task>SMC)							
Insula/Adjacent cortex – L	1347		−12	−32	18	4.40	0.027
Insula		279					
Superior temporal gyrus		80					
Rolandic operculum		451					
Postcentral gyrus		61					
Supramarginal gyrus		66					
Medial wall	1556		−8	−62	48	4.04	0.022
Precuneus – R		602					
Precuneus – L		646					
Middle cingulate gyrus – R		172					
Posterior cingulate gyrus – R		54					

Dorsal ACC, dorsal anterior cingulate cortex; L, left; R, right; FDR, false discovery rate; SMC, sensorimotor control.

1 Comparisons for the healthy controls (*N* = 14) and patients with schizophrenia (*N* = 14).

2 Identification of activation according to the Wake Forest University (WFU) Pickatlas.

3 Cluster extent.

4 Number of voxels within region identified by WFU Pickatlas; voxel size: 1.5 mm.

5 *x*, *y*, and *z* coordinates in MNI space for most significant voxel within the cluster.

6 FDR-corrected for cluster.

7 Cluster may extend into Presupplementary Motor Area, not recognized by WFU Pickatlas.

**Figure 7:**
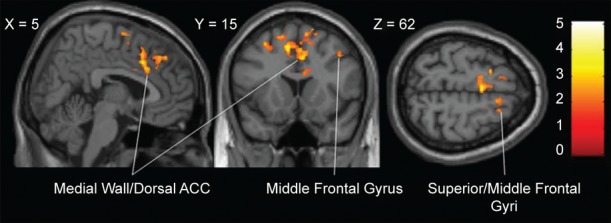
Between-group results for activation to hard>easy trials revealed an interaction between difficulty and group. For controls>consistent patients, the contrast is hard>easy; for consistent patients>controls, the contrast is easy>hard. *P* < 0.05, FDR-corrected. Conventions as in Figure 6.

Additional contrasts of interest were related to DD trial difficulty. Although the within-group analyses of activation to hard>easy trials were not significant in HC or in SZ, the reverse contrast of easy>hard trials revealed significant results in both groups (Table S3). HC exhibited activation in areas including the middle cingulate gyrus, superior parietal cortex, insula, and middle temporal cortex. SZ showed activation in the superior and middle frontal gyri, middle and posterior cingulate gyrus, inferior parietal cortex, and middle temporal cortex. Comparing groups for the difference in activation to easy versus hard trials (Fig. 7, Table 3) showed an interaction between group and difficulty in one large cluster that included lateral frontal regions such as the superior and middle frontal gyri, medial wall regions such as the dACC extending into the SMA/pre-SMA areas, and parietal locations such as inferior parietal lobule.

**Table 3:** Consistent patients and consistent controls: between-group fMRI results for trial difficulty^1^

Brain regions	Cluster	Voxels	*x*	*y*	*z*	*t*	*P*
Frontal cortex – R	6010		22	−2	54	5.06	<0.001
Superior frontal gyrus		297					
Middle frontal gyrus		259					
Precentral gyrus		275					
Frontal cortex – L							
Superior frontal gyrus		417					
Middle frontal gyrus		382					
Medial wall/Dorsal ACC							
Superior medial frontal gyrus – R		220					
Superior medial frontal gyrus – L		319					
Supplementary motor area – R		679					
Anterior cingulate gyrus – R		213					
Anterior cingulate gyrus – L		60					
Middle cingulate gyrus – R		35					
Middle cingulate gyrus – L		294					
Parietal cortex – L							
Inferior parietal lobule		326					
Postcentral gyrus		175					

Other conventions as in Table 2.

1 Differential activation between the healthy controls (*n* = 14) and patients with schizophrenia (*n* = 14). For controls>patients, contrast is hard>easy trials; for patients>controls, contrast is easy>hard trials.

**Figure 8:**
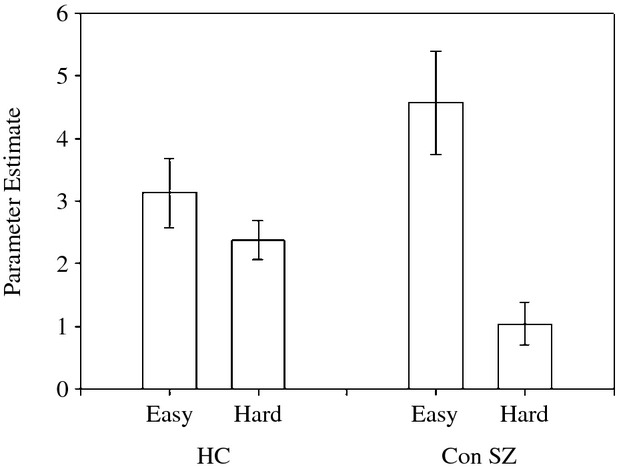
Mean (± standard error) parameters estimates extracted from the each participant's contrast maps for hard trials and easy trials using a functionally defined composite mask for the between-group results for hard versus easy trials. HC, healthy controls; Con SZ, consistent SZ.

In order to elucidate the interaction between group and difficulty, a composite mask was created of the significant between-group differences. Bar graphs of mean beta values for hard and easy trials versus baseline (Fig. 8) suggested that both HC and SZ had greater activation to the easy trials than to the hard trials in the region, with SZ exhibiting a greater difference between easy and hard trials.

#### Inconsistent SZ

**Figure 9:**
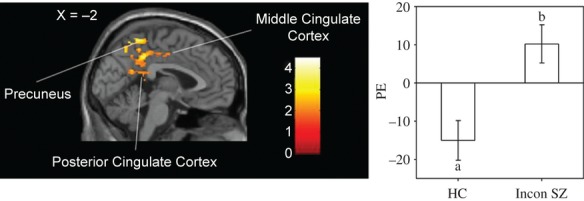
Between-group fMRI results for activation in inconsistent patients (*n* = 9) when compared with consistent controls (*n* = 14) to task>SMC trials for the largest, medial cluster activated. Left, the sagittal brain section shows greater activation occurred in inconsistent SZ when compared with consistent controls on the medial wall (medial wall/parietal/occipital cluster size 3048 of Table 4); in particular, in the precuneus and posterior and middle cingulate cortex. Voxel-level intensity threshold uncorrected *P* < 0.05, with a minimum cluster size to maintain a FDR = 0.05. No regions were more activated in the consistent controls than in the inconsistent SZ. Right, mean (± standard error) parameter estimates (PE) extracted for the functionally defined mask of group differences in inconsistent SZ (task>SMC) > consistent controls (task>SMC) for the same medial wall cluster. a, *P* = 0.043 versus 0; b, *P* = 0.076 versus 0.

In a within-group analysis (Table S5), limited activation in inconsistent patients during DD task versus SMC trials occurred in a small region in the left frontal cortex and in regions in the left parietal and occipital cortices. Table 4 shows the between-group comparisons of activation to DD task>SMC trials in inconsistent patients versus consistent controls and in inconsistent patients versus consistent patients. The inconsistent patients exhibited *greater* activation to the task than controls. The greater activation was in two clusters within posterior medial wall regions, such as the precuneus, posterior, and middle cingulate, and calcarine cortex (Fig. 9, left). To clarify the group difference, we extracted mean parameter estimates from these clusters. Results were similar in both clusters – there was significantly *decreased* activation in the consistent controls and marginally significant *increased* activation in inconsistent patients. Results for one of the clusters are plotted in Figure 9, right.

**Table 4:** Between-group results for activation to task>SMC trials^1^

Brain regions	Cluster	Voxels	*x*	*y*	*z*	*t*	*P*^3^
Inconsistent patients(task>SMC)>consistent controls(task>SMC)							
Medial wall/Parietal cortex	2213		32	−41	58	4.50	0.001
Middle cingulate gyrus – bilateral		56					
Precuneus – bilateral		902					
Superior parietal lobule – bilateral		345					
Medial wall/Parietal/Occipital cortex	3048		−4	−42	41	4.34	<0.001
Postcentral gyrus		83					
Precuneus – bilateral		652					
Middle cingulate gyrus – bilateral		544					
Posterior cingulate gyrus – bilateral		297					
Cuneus – right		251					
Calcarine area – bilateral		445					
Superior occipital gyrus		30					
Lingual gyrus – right		121					
Inconsistent patients(task>SMC)>consistent patients(task>SMC)							
Frontal/Parietal cortex – left	1713		−33	24	46	4.20	0.01
Superior frontal gyrus		443					
Middle frontal gyrus		391					
Superior medial frontal		164					
Supplementary motor area^2^		142					
Precentral gyrus		331					
Postcentral gyrus		44					

Other conventions as in Table 2. FDR, false discovery rate; SMC, sensorimotor control.

1 Between-group comparisons for the inconsistent patients (*N* = 9), consistent controls (*N* = 14), and consistent patients (*N* = 14). Neither consistent controls nor consistent patients exhibited greater activation than inconsistent patients to the task>SMC trials.

2 Cluster may also extend into the presupplementary motor area, not recognized by the WFU Pickatlas.

3 Voxel-level intensity threshold uncorrected, *P* < .05, with a minimum cluster size to maintain FDR = 0.05.

**Figure 10:**
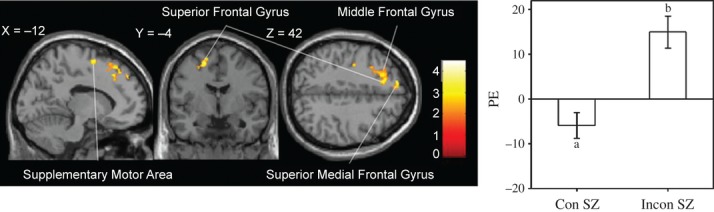
Left, the brain section shows between-group fMRI results for activation to task>SMC trials. More activation occurred in inconsistent SZ (*n* = 9) when compared with consistent SZ (*n* = 14) in the supplementary motor area, superior frontal, and superior medial frontal gyri. Right, mean (± standard error) parameter estimates (PE) extracted for the functionally defined mask of group difference for inconsistent patients (task>SMC) > consistent patients (task>SMC) for the cluster of Table 4 with the peak voxel at MNI coordinates −33, 24, and 46 in the middle frontal gyrus. a, *P* = 0.059 versus 0; b, *P* = 0.003 versus 0. Other conventions as in Figure 9.

For the DD task>SMC trial comparison of the two SZ groups, inconsistent patients showed greater activation than consistent patients in more frontal areas, such as the left superior and middle frontal gyri, and more medially, in the superior medial frontal gyrus and region of the pre-SMA (Strick et al. 1998; Zhang et al. 2012) (Table 4; Fig. 10, left). Comparison of mean parameter estimates for this cluster (Fig. 10, right) showed activation in the inconsistent patients and marginally significant deactivation in consistent SZ. The opposite contrast of activation to task trials>SMC trials in consistent patients greater than the inconsistent patients was not significant.

## Discussion

To our knowledge, this is the first study comparing the neural circuits engaged during a DD task in SZ. In order to avoid potential confounds related to differences in task performance, we compared fMRI activation in SZ and HC in groups exhibiting similar performance on a DD task. We found overall reduced activation to DD task trials compared with control trials in SZ, most notably in putative executive function and reward areas. On the other hand, SZ showed greater activation than controls in areas including the precuneus and posterior cingulate, which might suggest activation related to the engagement of compensatory mechanisms or reduced deactivation of regions belonging to the DMN, and in the insula, a region linked to emotional processing. Furthermore, consistent SZ had abnormal activation of lateral and medial frontal regions in relation to trial difficulty. Results of the contrasts including inconsistent SZ should be interpreted with caution because of their poor performance on the task. These results will be discussed in relation to previous studies of DD and of SZ.

### Behavioral results

Using a criterion level of *R*^2^ > 0.60 for inclusion in fMRI data analysis, our two main groups of interest were well matched on not only consistency (*R*^2^) but also rate of discounting, log(*k*), as well as percentage of Now choices across trial categories. Thus, differences in brain activation cannot be attributed to these differences in task performance. However, it should be noted that consistent SZ was generally slower in performing the task. Also, in contrast to HC, who took less time to respond to the easy than to the difficult trials, SZ took as much time to respond to both type of trials. Others have also observed abnormal RT modulation in SZ in response to task difficulty (Holcomb et al. 2004; Thakkar et al. 2010; Strauss et al. 2011). While inconsistent SZ had significantly higher log(*k*) than HC and consistent SZ, as discussed in the results section, the validity of the computed *k* values for this group is suspect.

The findings of no difference in rate of discounting between consistent SZ and consistent HC are different from those from our behavioral study (R. E. Weller, K. B. Avsar, J. E. Cox, M. A. Reid, D. M. White, A. C. Lahti, unpubl. ms.) carried out in the laboratory where we reported higher discounting rate in consistent SZ (*n* = 27) compared with HC (*n* = 21). For the imaging part of this project, we studied subgroups of SZ and HC that did not differ in task performance (*k* and *R*^2^) and provided useful data in the magnet.

### Imaging results

In consistent HC, as expected, the contrast of all DD task trials versus the SMC trials revealed activation in the ventral striatum, a region of the reward network, and executive function areas such as prefrontal, dACC, and inferior parietal cortex. The regions activated are similar to those of other fMRI studies of DD that used a comparable contrast (McClure et al. 2004; Hoffman et al. 2006; Monterosso et al. 2007; Bickel et al. 2009; Pine et al. 2009). The specific role each region contributes to DD is still controversial. McClure et al. (2004), for example, have argued that immediate or more impulsive and emotional choices are driven by the limbic system, whereas activation in lateral prefrontal, lateral orbitofrontal, and inferior parietal cortex occurs during all trials requiring a decision, and especially more difficult decisions.

The between-group analysis of all DD task trials versus SMC trials revealed that, in the face of matched performance, SZ had significantly less activation than HC in putative executive function areas, inferior frontal, dACC, and posterior parietal cortices; as well as in reward regions such as the ventral striatum and midbrain. The results of a recent meta-analysis (Minzenberg et al. 2009) have shown that, in general, executive tasks engage a distributed neural network, prominently including frontal (lateral and medial prefrontal cortex) and posterior parietal cortices and thalamus. The authors of this meta-analysis further report that SZ fail to engage this network to the same extent as HC and speculate that the findings are consistent with a disruption of a frontal-based cognitive control function. Our data concur with these results and extend them by additionally showing reduced engagement of regions of the reward system during decision making. SZ appear to lack an integrated neural response when making decisions. Abnormal modulations of ventral striatum/midbrain regions in SZ have been reported in association with various tasks taping into reward processes such as prediction error (Waltz et al. 2009; Koch et al. 2010), incentive monetary delay (Juckel et al. 2006a,b; Schlagenhauf et al. 2008), and aversive Pavlovian learning (Jensen et al. 2008). However, most of these studies have limited their analyses to regions of the ventral striatum or midbrain, leaving questions of integration with other networks unanswered. Further work will need to evaluate the specific contribution of cognitive control and reward networks to abnormalities such as those seen in this study. On the other hand, patients showed greater activation in a limited number of regions such as the precuneus, posterior cingulate gyrus, and insula extending into the frontal operculum and superior temporal gyrus. Perhaps these latter regions of activation served a compensatory role during performance of the DD task, allowing patients to perform similarly to controls in spite of showing blunted activation of putative executive function areas and reward areas. Greater activation in response to other (non-DD) tasks has also been reported in SZ when patient groups were matched on performance and interpreted as compensatory (Callicott et al. 2003; Avsar et al. 2011; Ettinger et al. 2011). On the other hand, the activated regions, the precuneus and posterior cingulate, are regions that are part of the so-called DMN (Gusnard et al. 2001; Raichle et al. 2001; Greicius et al. 2003; for review, see Cavanna and Trimble 2006). Recent work in fMRI supports the presence of two large-scale brain networks whose coupling is critical for optimal cognitive function: the “task-positive” network comprised of regions typically activated during task performance (dorsal ACC, lateral parietal, dorsolateral prefrontal), and the DMN comprised of regions activated when no task is performed and deactivated during a task (rostral ACC, precuneus, posterior cingulate cortex) (Fox et al. 2005). Our results could be interpreted as patients showing less task-induced deactivation in regions of the DMN, as others have with other tasks (Whitfield-Gabrieli et al. 2009; Jeong and Kubicki 2010). Interestingly, a lack of deactivation in precuneus and posterior cingulate was also observed during the DD task in the inconsistent SZ compared with HC, suggesting this finding is not related to task consistency.

The insula, a region consistently found abnormal in past structural and functional imaging studies in SZ (Wylie and Tregellas 2010; Palaniyappan and Liddle 2012), was more activated in consistent SZ compared with HC. The insula is part of the “somatic marker” network of brain areas showing increased activity during more emotional decisions (Damasio 1994). It is possible that performance of the DD task is emotionally more taxing for patients than for HC. Along with the ACC, the insula has recently been implicated in a network whose role is to enable the switch between the task positive and DMN (Menon and Uddin 2010). Reduced deactivation of regions of the DMN and abnormal insular/ACC activation might suggest disrupted coupling between brain networks.

We also compared the groups on activation based on task difficulty. On the hard>easy comparison, a contrast thought to tap more specifically into executive function, we did not identify any regions significantly activated in the HC or SZ groups, unlike the results of Marco-Pallares et al. (2010) and Kishinevsky et al. (2012). Interestingly though, in our study, the reverse contrast of easy>hard trials revealed widespread cortical activation in both groups, similar to results reported by Marco-Pallares et al. (2010). They found activation in multiple regions corresponding to our within-group results, such as the insula, middle cingulate gyrus, middle temporal cortex, and posterior parietal cortex. These authors characterized some of these regions as related to reward, which would apply to the insula activation in our study.

We identified an interaction between groups and trial difficulty in a large cluster prominently comprising the dACC/medial frontal cortex. In that region, both groups exhibited greater activation to the easy trials compared with the hard trails; however, the difference between easy and hard trials was larger in SZ. Because the function of the dACC/medial frontal cortex has been consistently linked to conflict monitoring (Kerns et al. 2005; Melcher et al. 2008; Reid et al. 2010), and SZ showed greater reaction time during the easy trials than HC, it is possible that, in SZ, the easy trials generated more conflict than in HC and, consequently, more activation in dACC/medial frontal cortex.

The greater frontal activation in inconsistent patients compared with consistent SZ may appear surprising, given that this region is often associated with higher cognitive functions and yet these patients showed poorer performance on the DD task. However, abnormal prefrontal cortex activation is one of the most replicated findings in SZ, with reports of hyper- and hypoactivation associated with fluctuating task difficulty and performance (Glahn et al. 2005). More activation in our inconsistent patients than consistent patients during the DD task may reflect inefficient processing during task performance.

### Study limitations

For the main fMRI contrasts done in this study we opted to match patients and HC based on performance which led us to exclude about a third of our patient population. This significantly limits the generalization of the results. The behavioral results from the inconsistent patients suggest that the task was too difficult and/or that the participants were not meaningfully engaged in the task. In future imaging studies, these patients could be compared with HC using a parametric equivalent of the DD task. All SZ in this study were on stable doses of antipsychotic medications, which may influence the BOLD fMRI signal (Roder et al. 2010). In addition, there was a trend level difference between the number of smokers in the consistent SZ group and the consistent HC group, and smoking negatively impacts the brain (Durazzo et al. 2006; Gallinat et al. 2007). However, two recent studies that took smoking into consideration found *no* group differences in DD between SZ and HC (MacKillop and Tidey 2011; Wing et al. 2012; but see Ahn et al. 2011). Performance on the RBANS was significantly impaired in consistent and inconsistent SZ compared with consistent HC. These cognitive deficits could contribute to differences in activation across a wide variety of tasks, including the present DD task. DD may also be influenced by a person's financial status. Given the financial circumstances of patients with chronic illness, this may be a psychological factor influencing behavior unrelated to symptoms associated with SZ. Finally, our small sample sizes did not allow us to pursue meaningful correlations with relevant factors, such as cognitive and clinical measures.

## Conclusions

Our results point to disruption of several neural networks during decision making, including executive, reward, default mode, and emotional, and suggest processes that are disturbed during decision making in SZ. In the face of matched behavior, executive and reward networks were less activated, while regions of the DMN that are usually deactivated during a task were more activated. Patients’ ability to perform the task may be perturbed because of disrupted coordination between normally inversely correlated networks. In addition, performance of easy trials may have generated more conflict in patients than in HC. Finally, the emotional regulation associated with making decision may be differently affected in patients than in HC.

In the face of matched performance, neural abnormalities have been identified that are likely associated with impaired decision making in SZ. Understanding the neural bases of abnormal DD in SZ could lead to interventions to improve decision making and goal-directed behavior in SZ.

## Supporting Information

**Data S1**. Supplemental material related to analyses included in this study.

**Data S2**. Supplemental results related to inconsistent SZ.

**Figure S1.** fMRI within-group activation to DD task>SMC trials for healthy controls (left) and consistent SZ (right). Results are shown on axial slices from ventral to dorsal; numbers are for MNI *z* coordinates. Voxel-level intensity threshold *P* < 0.001, uncorrected, with cluster-size threshold set to maintain FDR = 0.05.

**Table S1**. Tasks available during scanning.

**Table S2**. fMRI within-group results for consistent HC and consistent SZ for activation to task>SMC trials.

**Table S3**. fMRI within-group results for consistent HC and consistent SZ for activation to easy>hard trials.

**Table S4**. Demographic data and clinical and behavioral measures for imaging session participants.

**Table S5**. fMRI within-group results for inconsistent SZ from the analysis of activation to task>SMC trials.
