# Supplementary material for: An fMRI investigation of delay discounting in patients with schizophrenia
Source: Brain Behav. 2013 Apr 24;3(4):384–401. doi: 10.1002/brb3.135 (PMC3869680; doi:10.1002/brb3.135)
Supplement: Supplementary file 1 [file brb30003-0384-SD1.docx]

**SUPPLEMENTAL MATERIAL**

Avsar KB, Weller RE, Cox JE, Reid MA, White DM, Lahti AC. An fMRI Investigation of Delay Discounting in Patients with Schizophrenia and Controls Matched on Performance

**I. Supplemental material related to analyses included in paper**

A. Methods. Details about DD task administration.

Table I.1. Tasks available during scanning.

B. Results.

Table I.2. fMRI within-group results for consistent HC and consistent SZ for activation to task>SMC trials.

Table 1.3. fMRI within-group results for consistent HC and consistent SZ for activation to easy>hard trials.

Figure I.1. fMRI within-group results for consistent HC and consistent SZ for activation to task>SMC trials.

**II. Supplemental results related to inconsistent SZ**

1. Results in narrative form
2. Tables and figures

Table II.1. Demographic data, clinical and behavioral measures for imaging session participants.

Table II.2. fMRI within-group results for inconsistent SZ from the analysis of activation to task>SMC trials.

**I. A. Methods.**

Instructions to participants about DD task.

Participants were told that the purpose of the task was to choose between two hypothetical reward amounts. On each trial, one of the amounts would always be available immediately and the other choice would be available after a delay. An explicit instruction was also given to select based on their preference and not on what they might think someone else would want them to choose. They were also told that all choices were unrelated and to not attempt to plan ahead. Each decision was indicated by a button press on the corresponding side. For the sensorimotor control trials, they were instructed that they would see the same choices on the left and right sides and to choose arbitrarily.

Table 1 contains the ten tasks available for the scanning session with a trial k for each trial category. The task with a middle k (k_3_) most closely matching the *k* from the participant’s laboratory session would optimize responses from the participant so that he or she would choose an approximately equal number of immediate and delayed rewards during scanning. For example, a participant with *k* =.036 from the laboratory session would be assigned magnet task #8 with a middle k (k_3_) of 0.041.

| **Table I.1.** Tasks available during scanning. | | | | | | | | | | | |
| --- | --- | --- | --- | --- | --- | --- | --- | --- | --- | --- | --- |
| **Trial k** | **Reward Preference** | ***#1*** | ***#2*** | ***#3*** | ***#4*** | ***#5*** | ***#6*** | ***#7*** | ***#8*** | ***#9*** | ***#10*** |
| k_1_ (easy) | Immediate | 0.0004 | 0.0007 | 0.0007 | 0.001 | 0.0018 | 0.0025 | 0.0033 | 0.006 | 0.011 | 0.028 |
| k_2_ (hard) | Immediate | 0.001 | 0.0018 | 0.0025 | 0.0033 | 0.006 | 0.011 | 0.016 | 0.028 | 0.041 | 0.1 |
| k_3_ (hard) | Ambivalent | 0.0018 | 0.0025 | 0.0033 | 0.006 | 0.011 | 0.016 | 0.028 | 0.041 | 0.07 | 0.17 |
| k_4_ (hard) | Delayed | 0.0025 | 0.0033 | 0.006 | 0.011 | 0.016 | 0.028 | 0.041 | 0.07 | 0.1 | 0.25 |
| k_5_ (easy) | Delayed | 0.011 | 0.016 | 0.028 | 0.041 | 0.07 | 0.1 | 0.17 | 0.25 | 0.25 | 1 |
|  | | | | | | | | | | | |

I.B Results

| **Table I.2.** Within-group fMRI results for controls (n=14) and consistent patients with schizophrenia (n=14) for activation to task>SMC trials. | | | | | | |
| --- | --- | --- | --- | --- | --- | --- |
| **Controls** | | | | | | |
| **Brain Regions^1^** | **Cluster^2^** | **x^3^** | **y^3^** | **z^3^** | **t** | **P^4^** |
| **Frontal Cortex-Right** |  |  |  |  |  |  |
| Middle Frontal Gyrus | 281 | 51 | 42 | 14 | 8.86 | <0.001 |
| Inferior Frontal Gyrus | 239 | 52 | 12 | 29 | 7.65 | <0.001 |
| Inferior Frontal Gyrus | 103 | 31 | 22 | -1 | 5.58 | 0.001 |
| **Frontal Cortex-Left** |  |  |  |  |  |  |
| Inferior Frontal Gyrus | 638 | -48 | 16 | 29 | 8.79 | <0.001 |
| **Parietal/Occipital-Right** |  |  |  |  |  |  |
| Inferior Parietal Lobule | 190 | 32 | -47 | 39 | 9.01 | <0.001 |
| Middle Occipital Gyrus | 930 | 18 | -90 | -3 | 8.52 | <0.001 |
| **Parietal/Occipital-Left** |  |  |  |  |  |  |
| Inferior Parietal Lobule | 264 | -27 | -53 | 47 | 8.99 | <0.001 |
| Inferior Parietal Lobule | 92 | -42 | -42 | 48 | 7.46 | 0.002 |
| Middle Occipital Gyrus | 1282 | -32 | -84 | 0 | 10.48 | <0.001 |
| **Temporal Cortex-Left** |  |  |  |  |  |  |
| Inferior Temporal Gyrus | 184 | -46 | -54 | -9 | 6.57 | <0.001 |
| **Medial Wall/Dorsal ACC** |  |  |  |  |  |  |
| Medial Frontal Gyrus | 621 | 0 | 19 | 50 | 7.0 | <0.001 |
| Anterior Cingulate-R | 94 | 3 | 36 | 32 | 7.58 | 0.002 |
| Precuneus-R | 306 | 26 | -63 | 42 | 7.5 | <0.001 |
| Precuneus-L | 81 | -21 | -60 | 47 | 6.22 | 0.004 |
| **Thalamus/Basal ganglia** |  |  |  |  |  |  |
| Thalamus/Striatum | 45 | -6 | -29 | -3 | 6.1 | 0.034 |
| Thalamus/Striatum | 40 | 4 | -29 | -3 | 5.98 | 0.047 |
| **Cerebellum-Left** |  |  |  |  |  |  |
| Cerebellum | 57 | -8 | -77 | -37 | 7.48 | 0.016 |
| Cerebellum | 55 | -3 | -72 | -27 | 4.86 | 0.017 |
| **Consistent SZ** | | | | | | |
| **Frontal Cortex-Right** |  |  |  |  |  |  |
| Inferior Frontal Gyrus | 51 | 33 | 21 | 0 | 6.36 | 0.010 |
| **Parietal/Occipital-Right** |  |  |  |  |  |  |
| Middle Occipital Gyrus | 114 | 22 | -96 | 10 | 8.97 | <0.001 |
| **Parietal/Occipital-Left** |  |  |  |  |  |  |
| Middle Occipital Gyrus | 43 | -39 | -86 | -4 | 10.26 | 0.019 |
| Middle Occipital Gyrus | 323 | -26 | -87 | 0 | 9.22 | <0.001 |
| **Medial Wall** |  |  |  |  |  |  |
| Medial Frontal Gyrus | 54 | -6 | 19 | 45 | 6.28 | 0.009 |
| Cuneus | 40 | 14 | -95 | 8 | 7.35 | 0.023 |
| Precuneus | 73 | 33 | -71 | 35 | 12.71 | 0.002 |
| **Cerebellum-Left** |  |  |  |  |  |  |
| Cerebellum | 123 | -3 | -69 | -27 | 11.65 | <0.001 |
| ^1^Labeling of activation according to WFU Pickatlas.  ^2^Cluster Extent.  ^3^x, y and z coordinates in MNI space of most significant voxel within the cluster.  ^4^FDR-adjusted p for cluster. | | | | | | |

| **Table I.3.** Within-group fMRI results for controls (n=14) and consistent SZ (n=14) for activation to easy>hard trials. | | | | | | |
| --- | --- | --- | --- | --- | --- | --- |
| **Controls** | | | | | | |
| **Brain Regions** | **Cluster** | **x** | **y** | **z** | **t** | **P** |
| **Parietal/Occipital-Right** |  |  |  |  |  |  |
| Angular Gyrus | 1292 | 38 | -71 | 45 | 4.97 | 0.014 |
| **Parietal/Occipital-Left** |  |  |  |  |  |  |
| Superior Occipital Gyrus | 1402 | -16 | -81 | 30 | 6.65 | 0.011 |
| **Temporal Cortex-Right** |  |  |  |  |  |  |
| Middle Temporal Gyrus | 2124 | 64 | -29 | -3 | 7.26 | 0.001 |
| **Insula/Temporal Cortex-Left** |  |  |  |  |  |  |
| Middle Temporal Gyrus | 2396 | -66 | -32 | 30 | 5.75 | 0.001 |
| Insula | 2036 | -33 | -5 | 11 | 5.99 | 0.001 |
| Fusiform Gyrus | 1014 | -38 | -74 | -19 | 5.20 | 0.030 |
| **Medial Wall/Dorsal ACC** |  |  |  |  |  |  |
| Middle Cingulate Gyrus-R | 1217 | 8 | -30 | 38 | 4.85 | 0.015 |
| **Cerebellum-Right** | 1241 | 22 | -81 | -16 | 5.19 | 0.015 |
| **Cerebellum-Left** | 1030 | -15 | -75 | -40 | 6.70 | 0.03 |
| **Consistent SZ** | | | | | | |
| **Frontal Cortex-Right** |  |  |  |  |  |  |
| Middle Frontal Gyrus | 4178 | 28 | 18 | 48 | 5.12 | 0.001 |
| **Frontal Cortex-Left** |  |  |  |  |  |  |
| Superior Frontal Gyrus | 876 | -18 | 31 | 48 | 5.00 | 0.027 |
| Precentral Gyrus | 2047 | -14 | -11 | 33 | 6.09 | 0.001 |
| **Parietal/Occipital-Right** |  |  |  |  |  |  |
| Inferior Parietal | 1830 | 38 | -42 | 48 | 5.12 | 0.001 |
| Middle Occipital Gyrus | 1036 | 36 | -78 | 12 | 5.54 | 0.017 |
| **Temporal Cortex-Left** |  |  |  |  |  |  |
| Middle Temporal Gyrus | 900 | -45 | -48 | 8 | 3.87 | 0.026 |
| **Medial Wall/Dorsal ACC** |  |  |  |  |  |  |
| Middle Cingulate Gyrus-R | 1210 | 4 | -36 | 39 | 4.54 | 0.008 |
| Posterior Cingulate-R | 1584 | 21 | -59 | 5 | 4.91 | 0.002 |
| Cuneus-R | 911 | 3 | -81 | 15 | 5.13 | 0.026 |
| No activation survived the corrected threshold for hard>easy trials. Other conventions as in Table I.2. | | | | | | |

Controls

Consistent SZ


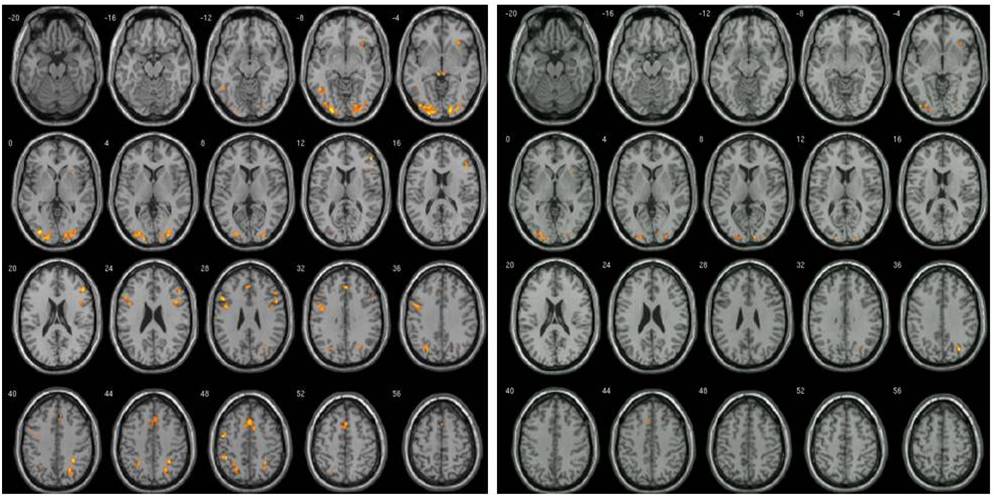


**Figure I.1.** fMRI within-group activation to DD task>SMC trials for healthy controls (left) and consistent SZ (right). Results are shown on axial slices from ventral to dorsal; numbers are for MNI z coordinates. Voxel-level intensity threshold P<.001, uncorrected, with cluster-size threshold set to maintain FDR=.05.

**II. Results for Inconsistent SZ.**

In an exploratory analysis, given the group’s poor task performance and smaller sample size (n=9), we also analyzed fMRI data from the inconsistent SZ for the contrast of activation to DD task versus SMC trials. Demographic data, clinical and behavioral measures for the inconsistent SZ are given in **Table I.1.** For comparison, data from the consistent controls and consistent SZ are also included.

Imaging Results. In a within-group analysis (**Table II.2**), limited activation in inconsistent patients during DD task versus SMC trials occurred in a small region in the left frontal cortex and in regions in the left parietal and occipital cortices.

| **Table II.1.** Demographic Data, Clinical and Behavioral Measures for Imaging Session Participants | | | | | |
| --- | --- | --- | --- | --- | --- |
| Variable | Consistent Controls  (n=14) | Consistent Patients  (n=14) | Inconsistent Patients  (n=9) | *F*_2,34_ | *P* |
| Age, years | 34 ± 3 | 37 ± 4 | 41 ± 3 | .74 | .48 |
| Gender | 8 Men; 6 Women | 10 Men; 4 Women | 7 Men; 2 Women | 2.78 | .096 |
| Parental SES^a^ | 6.46 ± 4.93 | 6.00 ± 1.24 | 8.50 ± 1.67 | .81 | .45 |
| Smoking | .27 ± .11^d^ | .59 ± .15^de^ | .81 ± .28^e^ | 2.53 | .10 |
| RBANS^b^ |  |  |  |  |  |
| Total Index | 95.69 ± 3.0^d^ | 77.93 ± 2.81^e^ | 73.88 ± 2.573^e^ | 23.43 | <.001 |
| Immediate Memory | 97.15 ± 3.09^d^ | 82.64 ± 3.58^e^ | 77.00 ± 5.67^e^ | 10.53 | .003 |
| Visuospatial | 96.62 ± 4.35^d^ | 80.50 ± 4.96^e^ | 77.63 ± 2.81^e^ | 6.99 | .013 |
| Language | 96.46 ± 4.06^d^ | 91.36 ± 1.90^d^ | 88.25 ± 4.10^d^ | 3.95 | .26 |
| Attention | 98.46 ± 4.58^d^ | 83.43 ± 4.35^e^ | 84.88 ± 2.89^de^ | 6.92 | .03 |
| Delayed Memory | 98.00 ± 1.73^d^ | 77.79 ± 5.15^e^ | 69.50 ± 6.56^f^ | 14.80 | .001 |
| BPRS |  |  |  | *F*_1,21_ |  |
| Total |  | 32.57 ± 2.60 | 31.67 ± 2.71 | .05 | .82 |
| Positive |  | 6.64 ± 1.06 | 5.56± 1.42 | .37 | .54 |
| Negative |  | 4.93 ± .61 | 4.67 ± .83 | .07 | .80 |
| Delay Discounting |  |  |  | *F*_2,34_ |  |
| Log_10_(*k)* | -1.91 ±.18^d^ | -1.70 ± .18^d^ | -.02 ± .24^e^ | 143.30 | <.001 |
| Imaging *R*^2^ | .92 ± .01^d^ | .91 ± .02^d^ | .26 ± .05^e^ | 20.74 | <.001 |
| Conventions same as in Table 2 except where noted.  ^a^Data not available for 4 consistent controls, 1 consistent patient and 2 inconsistent patients.  ^b^RBANS data not available for 1 healthy control and 1 inconsistent patient.  ^d,e,f^ Groups with different superscripts are significantly different. | | | | | |

| **Table II.2.** Within-group Results for Inconsistent Patients (n=9) for Activation to Task>SMC Trials. | | | | | | | |
| --- | --- | --- | --- | --- | --- | --- | --- |
| **Brain Regions** | **Cluster** | **Voxels** | **x** | **y** | **z** | **t** | **P** |
| **Frontal Cortex-Left** | 113 |  | -44 | 11 | 27 | 7.94 | <.001 |
| Inferior Frontal Gyrus |  | 110 |  |  |  |  |  |
| **Parietal Cortex-Right** | 54 |  | 27 | -60 | 50 | 6.00 | .019 |
| Superior Parietal Lobule |  | 28 |  |  |  |  |  |
| Precuneus |  | 25 |  |  |  |  |  |
| **Parietal Cortex-Left** | 49 |  | -28 | -54 | 44 | 8.86 | .023 |
| Inferior Parietal Lobule |  | 19 |  |  |  |  |  |
|  | 36 |  | -27 | -72 | 45 | 6.92 | .049 |
| Superior Parietal Lobule |  | 17 |  |  |  |  |  |
| **Occipital Cortex-Right** | 46 |  | 30 | -89 | 3 | 7.15 | .025 |
| Middle Occipital Gyrus |  | 46 |  |  |  |  |  |
| **Occipital Cortex-Left** | 36 |  | -4 | -98 | 5 | 8.65 | .049 |
| Cuneus |  | 34 |  |  |  |  |  |
|  | 91 |  | -15 | -93 | -4 | 8.39 | .001 |
| Calcarine Cortex |  | 42 |  |  |  |  |  |
| Inferior Occipital Gyrus |  | 20 |  |  |  |  |  |
| Lingual Gyrus |  | 17 |  |  |  |  |  |
| Conventions as in Table I.2. | | | | | | | |
